# Environmental Determinants of *Aedes albopictus* Abundance at a Northern Limit of Its Range in the United States

**DOI:** 10.4269/ajtmh.19-0244

**Published:** 2019-12-12

**Authors:** Pallavi A. Kache, Gillian Eastwood, Kaitlin Collins-Palmer, Marly Katz, Richard C. Falco, Waheed I. Bajwa, Philip M. Armstrong, Theodore G. Andreadis, Maria A. Diuk-Wasser

**Affiliations:** 1 Department of Ecology, Evolution, and Environmental Biology, Columbia University, New York City, New York;; 2 Department of Entomology, Virginia Polytechnic Institute and State University, Blacksburg, Virginia;; 3 Center for Vector Biology & Zoonotic Diseases, Connecticut Agricultural Experiment Station, New Haven, Connecticut;; 4 The Louis Calder Center—Biological Field Station, Fordham University, Armonk, New York;; 5 Bureau of Communicable Disease Control, New York State Department of Health, Albany, New York;; 6 Office of Vector Surveillance and Control, New York City Department of Health and Mental Hygiene, New York, New York

## Abstract

*Aedes albopictus* is a vector of arboviruses with high rates of morbidity and mortality. The northern limit of *Ae. albopictus* in the northeastern United States runs through New York state (NYS) and Connecticut. We present a landscape-level analysis of mosquito abundance measured by daily counts of *Ae. albopictus* from 338 trap sites in 12 counties during May–September 2017. During the study period, the mean number of *Ae. albopictus* caught per day of trapping across all sites was 3.21. We constructed four sets of negative binomial generalized linear models to evaluate how trapping methodology, land cover, as well as temperature and precipitation at multiple time intervals influenced *Ae. albopictus* abundance. Biogents-Sentinel (BGS) traps were 2.78 times as efficient as gravid traps and 1.49 times as efficient as CO_2_-baited CDC light traps. Greater proportions of low- and medium-intensity development and low proportions of deciduous cover around the trap site were positively associated with increased abundance, as were minimum winter temperature and March precipitation. The cumulative precipitation within a 28-day time window before the date of collection had a nonlinear relationship with abundance, such that greater cumulative precipitation was associated with increased abundance until approximately 70 mm, above which there was a decrease in abundance. We concluded that populations are established in Nassau, Suffolk, and New York City counties in NYS; north of these counties, the species is undergoing population invasion and establishment. We recommend that mosquito surveillance programs monitoring the northward invasion of *Ae. albopictus* place BGS traps at sites chosen with respect to land cover.

## INTRODUCTION

The global resurgence of mosquito-borne diseases highlights the importance of monitoring vector populations to quantify localized risk of arboviral transmission.^1–4^ In particular, the recent emergence of dengue virus (DENV), chikungunya virus (CHIKV), and Zika virus (ZIKV) can be attributed in part to the geographic expansion of *Aedes* spp. mosquitoes, including into temperate regions.^5^ Throughout the tropics, DENV, CHIKV, and ZIKV are transmitted to humans through the bite of the primary vector *Ae. aegypti* and, secondarily, *Ae. albopictus* (the Asian tiger mosquito).^6,7^ *Aedes albopictus* is a competent vector of these arboviruses and has been implicated as the primary vector in instances of autochthonous DENV infections in the United States and CHIKV in Europe, highlighting the importance of surveillance for this species, even in the absence of endemic diseases.^8–11^ We leverage vector surveillance data from the northeastern United States to understand how landscape and meteorological factors affect *Ae. albopictus* abundance at a northern limit of the species range.

Mosquito surveillance in the northeastern United States was largely initiated during the late 1990s and early 2000s, when state and local public health agencies established networks of mosquito collection sites to monitor potential outbreaks and epizootics of West Nile virus (WNV) and eastern equine encephalitis virus (EEEV).^12,13^ Since then, during May–October, programs commonly deploy CO_2_-baited CDC miniature light traps (CDC LTs) and gravid traps (GTs) at fixed locations. Given the historical emphasis on WNV and EEEV, traps are often placed in areas with suitable habitat for vectors of these arboviruses (e.g., areas with higher human population density for *Culex* sp. [WNV] and forested wetlands for *Culiseta* sp. [EEEV]). After the 2015–2016 ZIKV disease (ZVD) epidemic in Latin America and the Caribbean, there was a resurgence in efforts to investigate *Aedes* spp. (most notably, *Ae. aegypti* and *Ae. albopictus*).^4,14^ During 2016–2017, programs in the United States conducted enhanced trapping with Biogents-Sentinel (BGS) traps and viral testing of pools of *Aedes* spp. samples, with a focus on *Ae. albopictus* in the northeastern United States.

*Aedes albopictus* originated in East Asia, but now has established populations in countries across Asia, southern Europe, Africa, and the Americas, spanning tropical, subtropical, and temperate habitats.^5,15–18^ The species was first detected in the United States (Houston, TX) in 1985, likely introduced through the global tire trade.^19^ *Aedes albopictus* has since extended its distribution in the continental United States through repeated introduction events and range expansion.^9,20^ Ecological niche models indicate the potential for this mosquito to survive and reproduce as far north as Massachusetts.^21^ However, the eastern range of established *Ae. albopictus* populations remains constrained to southern New York state (NYS) and Connecticut.^22^

Climatic factors, including temperature and precipitation, are fundamental to *Ae. albopictus* introduction, survival, and persistence.^23–25^ Winter temperature has been cited as the most important factor in the distribution of *Ae. albopictus* at the northern limits of its range.^25–27^ Cold-acclimated, diapausing eggs have been shown to survive at low temperatures (−10°C) for up to 24 hours.^28^ However, longer durations of cold exposure decrease the hatching rate of mosquito eggs and survivorship of larvae.^30,31^ For temperate populations, *Ae. albopictus* is most active between the late spring and early fall. During this “growing season,” higher temperatures decrease egg, larval, and pupal development times.^24,31^ Specifically, the larval development time can range from 5.5 to 27 days (at 36°C and 15°C, respectively), whereas the pupal development time can range from 1.7 to 8.5 days (at 36°C and 15°C, respectively). Rainfall contributes to the accumulation of standing water in container habitats, which is critical to the development of juvenile life stages and facilitates flooding eclosion of previously deposited eggs.^32,33^ Therefore, short- and long-term variation in temperature and precipitation likely affect *Ae. albopictus* abundance.^26^

*Aedes albopictus* is most often found outdoors in peri-domestic environments, where it develops in natural and artificial containers and can feed on a wide range of animal hosts.^34–37^ The rapid spread of this species is associated with its ecological plasticity in breeding habitat, opportunistic feeding behavior, and tolerance of a wide temperature range.^38–40^ Temperate populations of *Ae. albopictus* produce diapausing eggs, allowing populations to persist in regions with seasonal temperature regimes.^7,13,22,41,42^ Gradients of urbanization have been shown to affect breeding site availability, larval densities, emergence rates, and survival time of *Ae. albopictus*, with urban environments often providing more suitable conditions than suburban or rural areas.^43,44^ In addition, studies have documented mosquito movement from endemic to new sites via highways and an association between *Ae. albopictus* density and distance to roads.^45,46^

To identify environmental factors associated with variation in *Ae albopictus* abundance, we used 2017 vector surveillance data from NYS and Connecticut counties with established *Ae. albopictus* populations. We evaluated land cover and meteorological factors with a strong mechanistic link to *Ae. albopictus* dispersal or population growth. Whereas previous models for the region have examined drivers of presence/absence or counts of *Ae. albopictus* aggregated annually, mosquito populations are known to have a high degree of spatial and temporal heterogeneity.^47^ By examining regional variation in land cover, temperature, and precipitation, as well as seasonal temperature and precipitation, our locally informed model can be used to identify jurisdictions with higher *Ae. albopictus* abundance and aid efforts to implement and evaluate mosquito surveillance, testing, and control.

## METHODS

### Study region.

**Figure 1.**
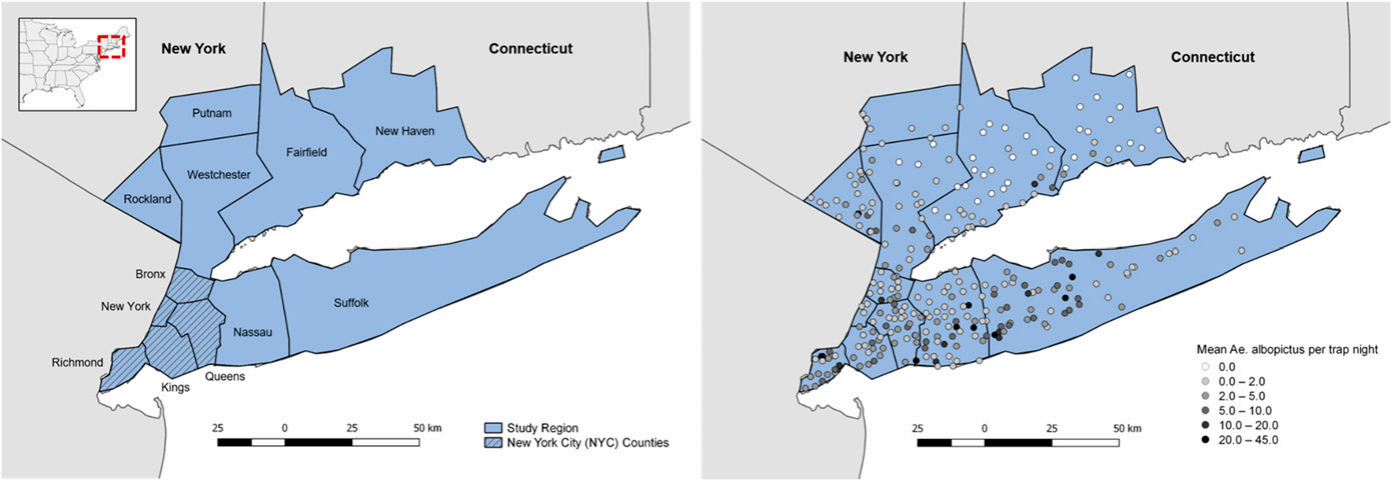
Map of the study region, New York state (NYS) and Connecticut (CT), United States. (**A**) Counties included in the study region. Ten counties in NYS (Suffolk, Nassau, Queens, Kings, Richmond, New York, Bronx, Westchester, Rockland, and Putnam) and two in Connecticut (Fairfield and New Haven). Five NYS counties make up New York City (NYC) and include Queens, Kings, Richmond, New York, and the Bronx; these correspond to the boroughs of Queens, Brooklyn, Staten Island, Manhattan, and the Bronx, respectively. (**B**) Distribution of trap sites (*N* = 332) throughout the study region. During May–September 2017, *Aedes albopictus* mosquitoes were present in approximately 89% of sites (*N* = 297). This figure appears in color at www.ajtmh.org.

**Figure 2.**
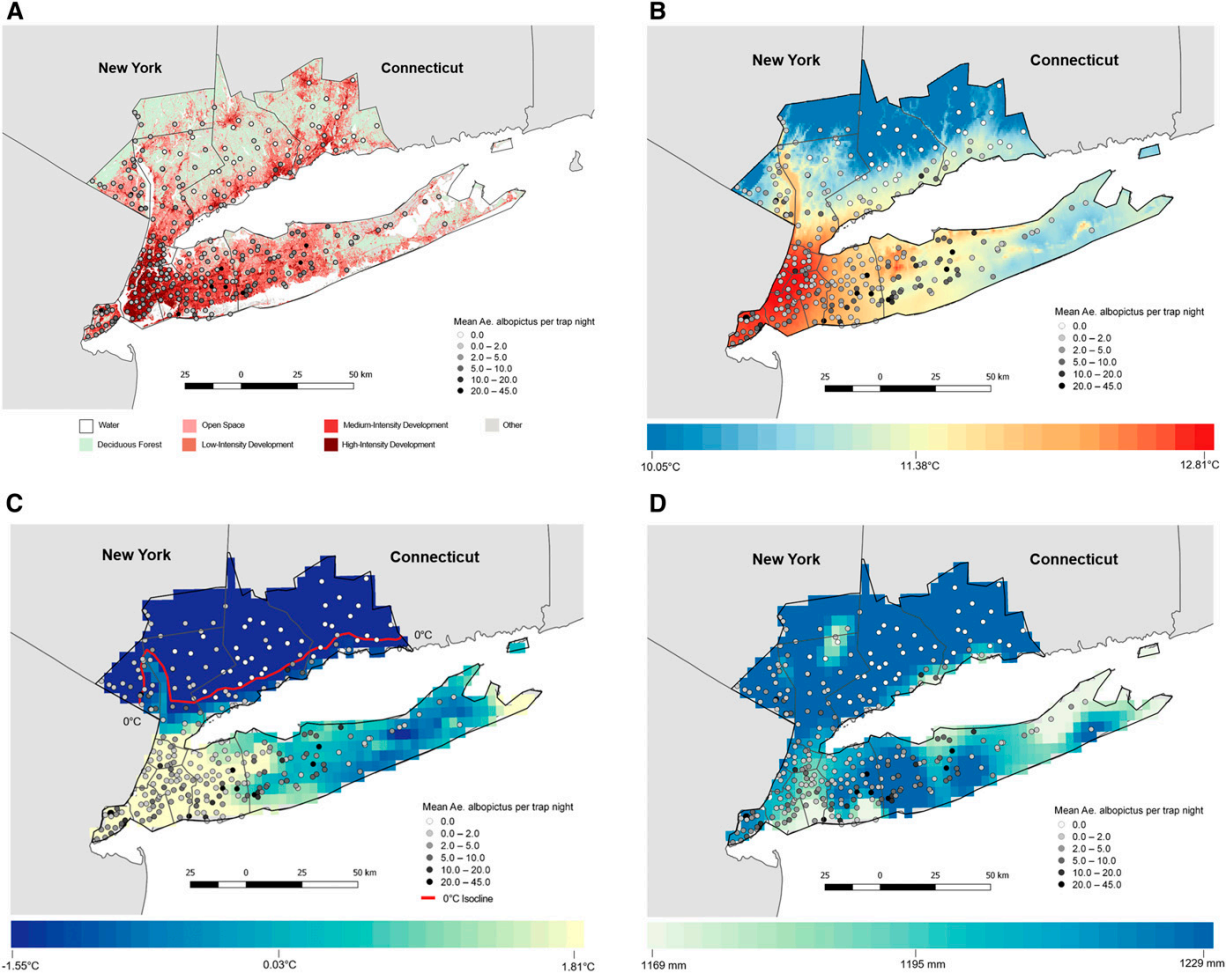
Land cover and climate across the study region, New York state and Connecticut (CT), United States. (**A**) Land cover classification across the study region. New York City (NYC) counties feature the greatest proportion of developed land (excluding water) (Bronx [79.44%], Kings [86.75%], New York [87.82%], Queens [88.61%], and Richmond [63.47%]), whereas Putnam (6.56%), Rockland (19.21%), and Fairfield, CT (23.04%) have the lowest. (Source: National Land Cover Database [NLCD, 2011]). (**B**) Mean annual temperature over the most recent three full decades (1981–2010). Temperatures are highest in NYC counties and lowest in the northern latitudes of Putnam, Fairfield, and New Haven counties. (Source: PRISM [Parameter-elevation Regression on Independent Slopes Model]. 30-year normals (1981–2010) at a 4-km spatial resolution. (**C**) Mean winter (December–February) temperature, 1981–2010. Again, temperatures are highest in NYC counties and the tip of Suffolk County, and lowest further inland from the Atlantic Coast (Rockland County) and the northern latitudes of Putnam, Fairfield, and New Haven counties. (**D**) Mean cumulative annual precipitation, 1981–2010. Across the study region, the mean cumulative annual precipitation was 1,202 mm; however, areas along the shoreline of Kings, Queens, Nassau, and Suffolk counties received less than the mean. This figure appears in color at www.ajtmh.org.

During 2017, *Ae. albopictus* surveillance was conducted for all counties in Connecticut and 13 counties in southeastern NYS, including New York City (NYC) and Long Island. We restricted data to 12 counties with established *Ae. albopictus* populations (Figure 1). The region varies considerably in human population density and land cover characteristics, including urban centers such as Manhattan, with 87.82% of county land (excluding water bodies) consisting of highly developed land cover, to rural localities such as Putnam County, with 6.56% developed land cover (excluding water bodies) (Figure 2A).

Mean annual temperatures range from 10.1°C to 12.8°C, with mean winter temperatures (December–February) ranging from −1.55°C to 1.81°C. Colder temperatures occur at more northern latitudes and further inland from the shoreline (Figure 2B and C). The region receives approximately 1,200 mm of precipitation per year (Figure 2D), with elevated precipitation between April and September.

### Mosquito trapping, collection, and processing.

Programs conducted mosquito surveillance during April–November 2017, although dates and frequency of trapping varied across counties. To minimize temporal biases, we restricted data to May 3–September 30, 2017.

A combination of CDC LTs, GTs, and BGS traps was deployed at 338 sites across the 12 counties. Comparative field trials have found the BGS to be more sensitive for monitoring *Aedes* spp. populations than CDC LTs and GTs.^48,49^ CO_2_-baited CDC LTs were hung at approximately 1.5 m and baited with 1–2 pounds of dry ice for a 24-hour trapping period. Gravid traps were baited with either a rabbit food pellet solution or hay-infused water. Biogents-Sentinel traps were placed on the ground and baited with BG-Lure, octenol lure, and/or dry ice to attract host-seeking mosquitoes. The number of hours that traps were deployed each trap day (i.e., “duration of trapping”) ranged from 12 to 24 hours according to resource-availability for each county; however, precise deployment times were not available. Traps were frequently placed at state parks or government-owned properties to minimize disturbance and increase ease of access. Captured specimens were transported to the laboratory on dry ice for species identification. Mosquitoes were sorted from other insect fauna and identified as *Ae. albopictus* by trained entomologists using a taxonomic guide.^50^

### Environmental data and processing.

We assessed the effects of land cover and road density within a circular buffer around each trap location on *Ae. albopictus* abundance. In addition, we evaluated the effects of seasonal and lagged weather variables on abundance (Table 1). Analyses were conducted in a projected geographic coordinate system, NY Long Island FIPS 3104 North American Datum of 1983/Universal Transverse Mercator zone 18N (NAD83/UTM zone 18N), using R 3.4.1 (R Core Team, Vienna, Austria) and ArcMap 10.2.1 (ESRI Inc., Redlands, CA).^51^

**Table 1:** Environmental variables assessed as covariates for *Aedes albopictus* abundance

Dataset	Class	Variable name	Description
National Land Cover Database (NLCD)*†	Developed	Open space	Areas with a mixture of some constructed materials, but primarily vegetation in the form of lawn grasses. Impervious surfaces account for less than 20% of total cover. Areas most commonly include large-lot single-family housing units, parks, golf courses, and vegetation planted in developed settings for recreation, erosion control, or aesthetic purposes.
		Low-intensity development	Areas with a mixture of constructed materials and vegetation. Impervious surfaces account for 20–49% of the total cover. Areas most commonly include single-family housing units.
		Medium-intensity development	Areas with a mixture of constructed materials and vegetation. Impervious surfaces account for 50–79% of the total cover. Areas most commonly include single-family housing units.
		High-intensity development	Highly developed areas where people reside or work in high numbers. Examples include apartment complexes, row houses, and commercial/industrial. Impervious surfaces account for 80–100% of the total cover.
	Forest	Deciduous forest	Areas dominated by trees generally greater than 5 m tall, and greater than 20% of total vegetation cover. More than 75% of the tree species shed foliage simultaneously in response to the seasonal change.
		Evergreen forest	Areas dominated by trees generally greater than 5 m tall, and greater than 20% of total vegetation cover. More than 75% of the tree species maintain their leaves all year. Canopy is never without green foliage.
	Water	Open water	Areas of open water, generally with less than 25% cover of vegetation or soil.
	Wetlands	Woody wetlands	Areas where forest or shrubland vegetation accounts for greater than 20% of vegetative cover, and the soil or substrate is periodically saturated with or covered with water.
		Emergent herbaceous wetlands	Areas where perennial herbaceous vegetation accounts for greater than 80% of vegetative cover, and the soil or substrate is periodically saturated with or covered with water.
	Planted/cultivated	Pasture/hay	Areas of grasses, legumes, or grass–legume mixtures planted for livestock grazing or the production of seed or hay crops, typically on a perennial cycle. Pasture/hay vegetation accounts for greater than 20% of total vegetation.
	Other	Other	Land cover classes found in small proportions throughout the study region included barren land, mixed forest, shrub/scrub, and cultivated crops.
Topologically Integrated Geographic Encoding and Referencing/Line (TIGER)‡		Road density	The density of primary and secondary roads within a 200-m radius of each trap location (m/m^2^). Primary roads are generally divided, limited-access highways within the interstate highway system or under state management, distinguished by the presence of interchanges. These highways are accessible by ramps and may include some toll highways. Secondary roads are main arteries, usually in the U.S. highway, state highway, and/or county highway system. These roads have one or more lanes of traffic in each direction, may or may not be divided, and usually have intersections with many other roads and driveways.
Parameter-elevation Regression on Independent Slopes Model (PRISM)§	Temperature	Mean winter temperature	Mean monthly temperature between December 2016 and February 2017 (°C).
		Minimum winter temperature	Minimum temperature between December 2016 and February 2017 (°C).
		Mean growing season temperature	Mean monthly temperatures of the *Aedes albopictus* population–growing season, between April and September 2017 (°C).
	Precipitation‖	Cumulative precipitation	Cumulative precipitation between January and December 2017 (mm).
		Cumulative precipitation during growing season	Cumulative precipitation between April and September 2017 (mm).
		January precipitation	Cumulative precipitation between January 01 and January 31, 2017 (mm).
		February precipitation	Cumulative precipitation between February 01 and February 28, 2017 (mm).
		March precipitation	Cumulative precipitation between March 01 and March 31, 2017 (mm).
		April precipitation	Cumulative precipitation between April 01 and April 30, 2017 (mm).
		Trap day precipitation	Precipitation on the date that the trap was set (mm).

* National Land Cover Database is a 16-class land cover database at a 30-m resolution (2011). U.S. Geological Survey and U.S. Department of the Interior.^52^

† Definitions provided for land cover classes that are included in the study region (see Supplemental Figure S1).

‡ Polyline shapefile of the U.S. road network (2017). U.S. Census Bureau, Department of Commerce.^54^

§ Regression-based dataset that generates repeatable estimates of daily, monthly, and annual temperatures at a 4.0-km spatial resolution (2017). Parameter-elevation Regression on Independent Slopes Model Climate Group, Oregon State University^78^

**Figure 3.**
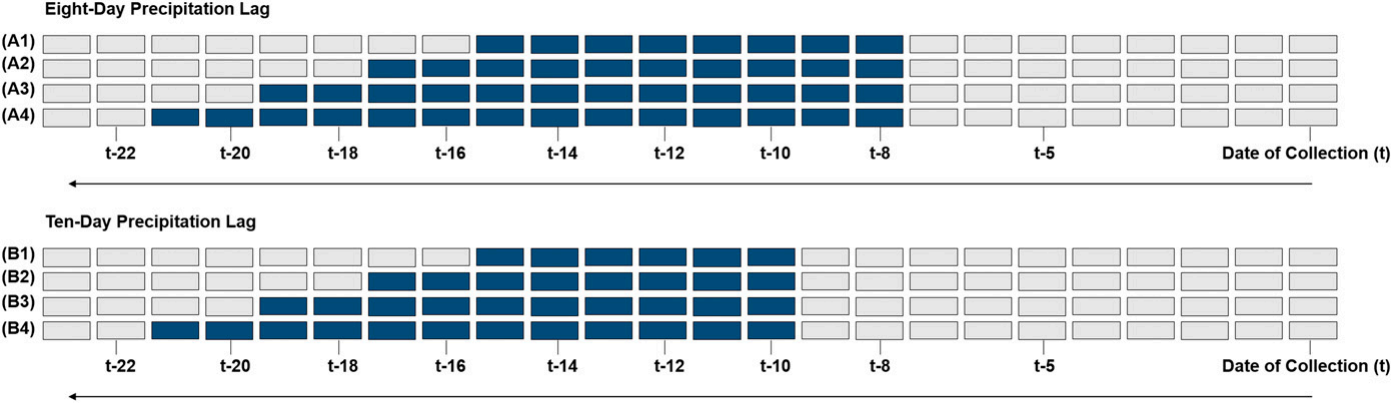
Schematic for assessing lagged precipitation as predictors of *Aedes albopictus* abundance. This schematic illustrates how we constructed precipitation predictor variables at two time lags and across multiple time windows to assess an association with *Ae. albopictus* abundance. We developed multiple time lags/windows to consider precipitation across a range of potential larval and pupal development times before capturing adults on the date of collection. Each row (e.g., A1–A4 and B1–B4) shows a unique time window in relation to the date of collection (*t*). Each gray box represents a day that precipitation information could have been included in each time window from the date of collection (*t*), up to 23 days before (*t*-23). Colored boxes indicate the days that were included for each unique time window. (**A**) We calculated mean and cumulative precipitation (millimeters) across 8-day (A1), 10-day (A2), 12-day (A3), and 14-day (A4) time windows beginning 8 days before the date of collection. In addition, we calculated values across 16-day, 18-day, 20-day, 22-day, and 24-day time windows (not shown). (**B**) We calculated the mean and cumulative precipitation (millimeters) across 8-day (B1), 10-day (B2), 12-day (B3), and 14-day (B4) time windows beginning 10 days before the date of collection. In addition, we calculated values across 16-day, 18-day, 20-day, 22-day, and 24-day time windows (not shown). In total, we assessed 16 time-lagged precipitation covariates. This figure appears in color at www.ajtmh.org.

‖ Excludes lagged precipitation predictors (see Figure 3).

#### Landscape variables.

Land cover data were collected from the National Land Cover Database (NLCD) 2011.^52^ This raster product contains 16 land cover classifications at a 30-m spatial resolution. To determine the most appropriate scale at which land cover metrics affect *Ae. albopictus* abundance, we evaluated multiple buffer sizes around each trap site. We calculated the percentage of each land cover class within circular buffers with 100-, 200-, 300-, 400-, and 500-m radii around each trap coordinate (R package “raster”).^53^ These buffer sizes were selected to capture a range of potential spatial scale effects around the maximum documented flight range of the mosquito in temperate regions (∼200–300 m).^53^ To determine road density around each trap site, we calculated the number of meters of primary and secondary road contained within a 200-m buffer of each trap (ArcGIS, “line density” toolset).^54^

#### Temperature and precipitation variables.

We derived predictor variables at multiple time scales, including monthly minimum and mean temperatures during each winter month (December 2016, January 2017, and February 2017), minimum and mean temperatures across winter months (December 2016–February 2017), growing season temperature (April–September 2017), annual precipitation, precipitation in each month before trapping (from January to April 2017), lagged precipitation up to 30 days before trap placement (Figure 3), and precipitation on the day of trap placement. We obtained meteorological data from Parameter-elevation Regression on Independent Slopes Model (PRISM) (R package “prism”). PRISM is a regression-based dataset that uses point data, a digital elevation model, spatial datasets, and subject-matter expert parameterization to generate repeatable estimates of daily, monthly, and annual temperatures at a 4.0-km spatial resolution.^55^

### Model development.

We constructed four sets of models to evaluate the effects of trapping methodology (Models 1 and 2), landscape patterns (Model 3), as well as landscape and weather patterns on *Ae. albopictus* abundance (Model 4).

We fit counts of *Ae. albopictus* trapped per day to a negative binomial generalized linear model (GLM) with a log link function to account for non-normality and overdispersion (R packages “MASS,” “vcd”). To account for variation in the hours of trap deployment (i.e., sampling effort), we included duration of trapping as an offset term (e.g., 12, 19, and 24 hours). All predictors were standardized by subtracting their mean and dividing by the SD.

We used an Akaike information criterion (AIC) model selection procedure to select the best fit and the most parsimonious model, where predictors with the lowest AIC values were considered for multivariable analyses. We tested all combinations of land cover predictor variables (Model 3) and land cover, temperature, and precipitation variables (Model 4).^56–58^ We assessed the AIC for each candidate model using the following equation:

AIC=−2⁡log(Likelihood)+2K, (1)

where *K* is the number of parameters in the model. The preferred candidate model has the lowest AIC, balancing model fit with parsimony. We then used a model-averaging approach, to incorporate information across multiple top-ranked models (ΔAIC < 2 of the best-performing model), and calculated Akaike weights (wAICs) for each model (Supplemental Methods). We calculated averaged parameter and standard error (SE) estimates for each predictor.^59^ Finally, we determined the relative importance (RI) for each predictor; this value ranges from 0 to 1 and shows the sum of the wAIC in each model where the predictor is present.

We conducted nonparametric Spearman’s rank order correlation tests to measure the strength of association between predictor variables and the variance inflation factor (VIF) for multicollinearity (R package “base”).

### Spatial autocorrelation.

Spatial autocorrelation indicates whether values for sampled locations nearby one another are more similar than values for sampled locations that are more distant. To evaluate if *Ae. albopictus* abundance was spatially autocorrelated, we developed a semivariogram model using residuals of the best-performing models for Models 3 and 4 (ArcGIS, “incremental spatial autocorrelation” toolset). The tool runs the global Moran’s *I* statistic for incremental distances, measuring the intensity of spatial clustering for each distance through a returned *z*-score (Supplemental Methods).

With these distances, we created autocovariate terms for Models 3 and 4 to account for the spatial dependency detected through semivariogram modeling.^60,61^ We tested two autocovariate terms for each model by creating neighbors for all trap sites based on threshold distances (i.e., distance band weights), where threshold distances were derived from the first-peak *z*-score or maximum-peak *z*-score. Spatial weights were row-standardized, where each neighbor weight for a site was divided by the sum of all neighbor weights for that site (R package “spdep”). Finally, we estimated the global Moran’s *I* of the residuals of the best-performing models for Models 3 and 4 to assess the reduction in spatial autocorrelation upon inclusion of first peak or maximum peak autocovariate terms (R package “spdep”).

### Spatial cross-validation.

To evaluate the final model (Model 4), we conducted spatially buffered leave-one-out cross-validation (LOOCV).^62,63^ Leave-one-out cross-validation uses a single observation from the original dataset as validation (i.e., testing) data, with the remaining observations kept as the training set; the analysis is repeated so that every observation in the original dataset is used once in the validation data.^64^ Traditional LOOCV does not consider spatial nonindependence, potentially resulting in artificially small error estimates and inflated estimates of model performance, even after inclusion of an autocovariate term.^65^ Therefore, we split the data into training and validation sets such that for each point left out for evaluation, all observations that fell within the range of spatial autocorrelation for that point were also removed. Remaining observations were used as the training dataset for the GLM, and predictions were made for the removed observation (i.e., validation set). We assessed the root mean square error (RMSE) of the squared prediction errors, where the prediction error was defined as the difference between the observed and predicted values at each cross-validation measurement.

## RESULTS

### Descriptive and spatial statistics.

In total, we considered 30,943 mosquitoes trapped across 338 trap sites during May 3–September 30. *Aedes albopictus* was present at 297 sites (89.46%). The mean distance between any one trap site and all others was 53.61 km, whereas the mean distance to the nearest site was 2.37 km. The number of mosquitoes trapped on any given day ranged from 0 to 194, with a mean of 3.21 (SD = 8.96). Analyses did not suggest that fewer hours of trapping impacted the mean number of *Ae. albopictus* collected (Supplemental Table S1).

### Landscape and meteorological drivers.

The majority of traps were placed in areas with a high proportion of developed land cover (mean proportion of developed land cover within a 500-m buffer of all traps combined [excluding water bodies] = 67.90%; SD = 5.46%). This stands in contrast to the heterogeneous land cover composition of the study region (mean proportion of developed land cover across the study region [excluding water bodies] = 40.67%; SD = 23.53%) (Supplemental Figure S1).

In bivariate models to identify the buffer size, open space and developed land cover classes had the best fit (lowest AIC) within 100- to 300-m buffers of each trap coordinate, whereas deciduous cover had the best fit at 500 m. At optimum buffer sizes, open spaces and deciduous cover were negatively associated with *Ae. albopictus* abundance, whereas developed land cover classes were positively associated with *Ae. albopictus* abundance (Supplemental Table S1). In addition, higher mean growing season temperatures were negatively associated with *Ae. albopictus* abundance, whereas higher mean winter temperatures and March precipitation were positively associated with abundance. When assessing precipitation in the weeks before trapping, we detected a nonlinear relationship between lagged precipitation and *Ae. albopictus* abundance, and created a quadratic term for this predictor. The best fit was over a 20-day time window, beginning 8 days before the date of collection (8-day lag) (exploratory data not shown).

### Statistical models.

#### Trap capture efficiency.

Detection of *Ae. albopictus* varied significantly with the trap type, accounting for land cover surrounding the trap and duration of trapping (Table 2, Model 1). Biogents-Sentinel traps were 2.78 times more efficient than GTs (incidence rate ratio [IRR] 95% CI: 2.50, 3.13; *P*-value: < 0.001) and 1.49 times more efficient than CDC LTs (IRR 95% CI: 1.37, 1.67; *P*-value: < 0.001) (Model 1).

**Table 2:** Effect of trapping methodology on *Aedes albopictus* detection

Model	Trapping method	Number of trap days (*N* = 9762)*	IRR	IRR 95% CI		Beta	Standard error	*Z*-score	*P*-value
1	BGS	4,091	Referent	–	–	–	–	–	–
	GT	2,296	0.36	0.32	0.40	−1.03	0.06	−17.35	< 0.001
	CDC LT	3,375	0.67	0.60	0.73	−0.41	0.05	−8.36	< 0.001
2	BGS: BG-Lure	3,512	Referent	–	–	–	–	–	–
	BGS: BG-Lure + octenol bait	302	1.34	1.07	1.69	0.29	0.12	2.52	0.01
	BGS: BG-Lure + octenol bait + CO_2_	275	5.94	4.70	7.62	1.78	0.12	14.56	< 0.001
	GT: hay infusion	1,531	0.65	0.57	0.74	−0.44	0.07	−6.70	< 0.001
	GT: rabbit pellet infusion	675	0.17	0.14	0.21	−1.76	0.11	−16.43	< 0.001
	CDC LT: CO_2_	3,375	0.91	0.82	0.99	−0.10	0.05	−2.02	0.04

BGS = Biogents-Sentinel trap; CDC LT = CDC light trap; GT = gravid trap; IRR = incidence rate ratio. This table presents the effect of mosquito-trapping methodology on *Ae. albopictus* abundance. Model 1 examines the efficiency of the BGS trap compared with the GT and CDC LT, accounting for land cover† and the duration of trapping. Model 2 examines the trapping efficiency of the BGS trap–baited BG-Lure compared with five other trapping methodologies, accounting for land cover class† and the duration of trapping. Efficiency is measured by the IRR, which provides a ratio of the number of *Ae. albopictus* detected per trap day for a given trapping method in relation to the referent trapping method. We obtain the IRR by exponentiating the beta regression coefficient (inverse results are presented in the Results section to show the IRR of the Referent in relation to other trapping methods).

* Total number of trap days is given by $\overset{n=332}{\underset{i=1}{\sum }}\left[{\left(\mathrm{Number}\;\mathrm{of}\;\mathrm{Traps\times Number}\;\mathrm{of}\;\mathrm{Days}\;\mathrm{of}\;\mathrm{Trapping}\right)}_{x_{1}}+\ldots +{\left(\mathrm{Number}\;\mathrm{of}\;\mathrm{Traps\times Number}\;\mathrm{of}\;\mathrm{Days}\;\mathrm{of}\;\mathrm{Trapping}\right)}_{x_{j}}\right],\;$where *i* indicates the number of trap sites and *j* indicates the trapping methods used per site.

† Land cover classes (with buffer sizes in meters): open space (300 m), low-intensity development (100 m), medium-intensity development (200 m), high-intensity development (200 m), deciduous vegetation (500 m), and woody wetland (500 m).

When examining the type of bait, BGS traps with BG-Lure alone were less efficient than BGS traps with BG-Lure enhanced with octenol bait and/or CO_2_ (Table 2, Model 2). However, BGS traps with BG-Lure alone were still more efficient than GTs and CDC LTs. Biogents-Sentinel traps with BG-Lure were 1.54 times as efficient as GTs baited with hay infusion (95% CI: 1.35, 1.75; *P*-value: < 0.001), 5.88 times as efficient as GTs baited with rabbit pellet infusion (95% CI: 4.76, 7.14; *P*-value: < 0.001), and 1.10 times as efficient as CDC LTs with CO_2_ (95% CI: 1.01, 1.22; *P*-value: 0.04).

#### Landscape drivers of *Ae. albopictus* abundance.

We constructed 256 models based on all combinations of land cover predictor variables, fixing trap type, and duration of trapping (offset) such that they were present in all models. Two models (Models 3A–3B) were considered best performing (ΔAIC < 2) and within 95% of the wAICs (Supplemental Table S2). Both had similar model weights (Model 3A, wAIC = 0.51; Model 3B, wAIC = 0.49). The best-performing model, Model 3A, included the proportion of low- and medium-intensity development (positive association), open space and deciduous cover (negative association), and road density as a quadratic term. All predictors had VIF scores < 3.0, indicating the variables did not exhibit multicollinearity. A global Moran’s *I* analysis indicated that autocovariate terms developed with distances of either the first-peak (17,074 m) or maximum-peak *z*-scores (36,392 m) of the semivariogram model did not remove spatial autocorrelation of the residuals entirely. However, assessing Model 3A, the first-peak *z*-score term resulted in a greater model fit and lower degree of spatial autocorrelation (ΔAIC = 1,003; *I* score = 0.26) compared with the maximum peak (ΔAIC = 1,030; *I* score = 0.28).

In the averaged model (Model 3), accounting for all other variables, we found that for every 1% increase in the proportion of low-intensity development within 100 m of the trap, the number of *Ae. albopictus* increased by a factor of 3.82 (scaled β estimate ± SE = 0.21 ± 0.02; *P*-value < 0.01); for every 1% increase in the proportion of medium-intensity development within 200 m, the number of mosquitoes increased by a factor of 4.75 (scaled β estimate ± SE = 0.27 ± 0.03; *P*-value < 0.01). By contrast, accounting for all other variables, a 1% increase in the proportion of deciduous cover within 500 m of the trap was associated with a decrease in abundance by a factor of 0.16 (scaled β estimate ± SE = −0.39 ± 0.03; *P*-value < 0.01); for every 1% increase in the proportion of open space within 300 m, the abundance decreased by a factor of 0.67 (scaled β estimate ± SE = −0.08 ± 0.03; *P*-value < 0.01). The RI for all variables was 1.0, with the exception of high-intensity development within 200 m of the trap (scaled β estimate ± SE = 0.03 ± 0.02; *P*-value = 0.16; RI = 0.51) (Table 3).

**Table 3:** Landscape drivers of *Aedes albopictus* detection

Model	Model-averaged coefficients	Estimate	95% CI		*P*-value	RI
3	Intercept	−2.90	−2.98	−2.81	< 0.01	1.0
	Autocovariate	0.32	0.30	0.34	< 0.01	1.0
	Gravid trap	−1.30	−1.41	−1.19	< 0.01	1.0
	CDC light trap	−0.87	−0.96	−0.77	< 0.01	1.0
	Open space: 300-m buffer	−0.08	−0.13	−0.03	< 0.01	1.0
	Low-intensity development: 100-m buffer	0.21	0.16	0.25	< 0.01	1.0
	Medium-intensity development: 200-m buffer	0.27	0.22	0.33	< 0.01	1.0
	High-intensity development: 200-m buffer	0.03	−0.01	0.09	0.48	0.51
	Deciduous forest: 500-m buffer	−0.38	−0.44	−0.31	< 0.01	1.0
	Road density	−0.60	−0.72	−0.47	< 0.01	1.0
	Road density^2^	0.65	0.52	0.78	< 0.01	1.0

RI = relative importance. This table presents the multi-model inferred averaged model. The 95% CI of the estimates indicate an effect on the detection of *Ae. albopictus* when the CI does not include zero (*P*-value < 0.05). The RI of a predictor variable (i.e., the probability of a variable being among the best-fitting models) was equivalent for all variables excluding the proportion of high-intensity development within a 200-m buffer.

#### Landscape and meteorological drivers of *Ae. albopictus* abundance.

**Figure 4.**
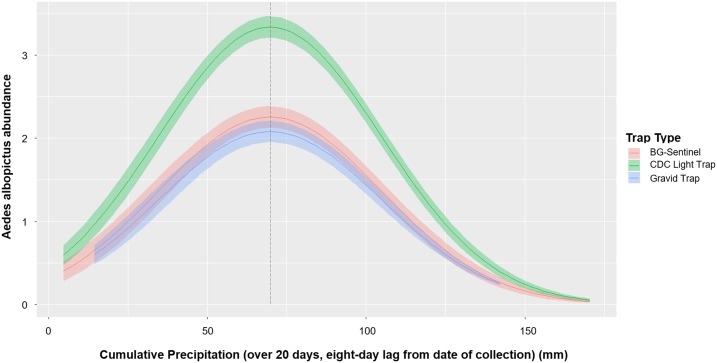
Predicted *Aedes albopictus* abundance as a function of lagged cumulative precipitation. Predicted *Ae. albopictus* abundance as a function of the cumulative precipitation over 20 days, with an eight-day lag from the date of collection (Figure 3), based on Model 4. All predictor variables for Model 4 were held at their mean values and the duration of trapping was assumed to be 24-hours, while we allowed cumulative precipitation to vary over its range. We found a quadratic relationship, with precipitation greater than 68.18 mm associated with decreased *Ae. albopictus* abundance. This figure appears in color at www.ajtmh.org.

We built 4096 models, based on combinations of meteorological predictor variables, fixing Model 3A predictor variables. Two models (Models 4A–4B) had ΔAIC values < 2 and were within 95% of the wAICs (Supplemental Table S3). In the averaged model (Model 4), the cumulative precipitation in a 20-day time window, with an 8-day lag, had the strongest effect on *Ae. albopictus* abundance (Table 4). We found a quadratic relationship best predicted *Ae. albopictus* abundance, with a positive association until precipitation in this time window reached 68.18 mm, followed by a negative association (Figure 4). Accounting for all other variables, for every 1°C increase in minimum winter temperature, the abundance of *Ae. albopictus* increased by a factor of 1.12 (scaled β estimate ± SE = 0.19 ± 0.04; *P*-value < 0.01); in addition, for every 1 mm increase in March precipitation, the abundance increased by a factor of 1.03 (scaled β estimate ± SE = 0.26 ± 0.03; *P*-value < 0.01).

**Table 4:** Landscape and meteorological drivers of *Aedes albopictus* detection

Model	Model-averaged coefficients	Estimate	95% CI		*P*-value	RI
4	Intercept	−2.77	−2.86	−2.67	< 0.01	1.0
	Autocovariate	0.26	0.24	0.29	< 0.01	1.0
	Gravid trap (GT)	−1.38	−1.49	−1.27	< 0.01	1.0
	CDC light trap (CDC LT)	−0.86	−0.96	−0.77	< 0.01	1.0
	Open space: 300-m buffer	−0.12	−0.17	−0.06	< 0.01	1.0
	Low-intensity development: 100-m buffer	0.17	0.12	0.21	< 0.01	1.0
	Medium-intensity development: 200-m buffer	0.26	0.21	0.31	< 0.01	1.0
	High-intensity development: 200-m buffer	0.04	−0.01	0.09	0.86	0.46
	Deciduous forest: 500-m buffer	−0.35	−0.42	−0.29	< 0.01	1.0
	Road density	−0.62	−0.75	−0.50	< 0.01	1.0
	Road density^2^	0.63	0.50	0.75	< 0.01	1.0
	Minimum winter temperature	0.19	0.11	0.26	< 0.01	1.0
	March precipitation	0.26	0.21	0.32	< 0.01	1.0
	Lagged precipitation (eight-day lag, 20-day sum*)	1.29	1.11	1.47	< 0.01	1.0
	Lagged precipitation^2^	−1.56	−1.74	−1.37	< 0.01	1.0

This table presents the multi-model inferred averaged model. The 95% CI of the estimates indicate an effect on the detection of *Ae. albopictus* when the CI does not include zero (*P*-value < 0.05). The RI of a predictor variable (i.e., the probability of a variable being among the best-fitting models) was equivalent for all variables excluding the proportion of high-intensity development within a 200-m buffer.

* Cumulative amount of rain in a 20-day time window beginning at the time step *t*-8, where *t* indicates the date of collection (see Figure 3).

Global Moran’s *I* analysis testing autocovariate terms developed with the distance of the first-peak (17,735 m), and the maximum-peak z-scores (37,501 m) of the semivariogram model did not remove spatial autocorrelation of the residuals entirely. However, assessing Model 4A, the first-peak *z*-score term resulted in a greater model fit and lower degree of spatial autocorrelation (ΔAIC = 451; *I* score = 0.22) compared with the maximum-peak (ΔAIC = 455; *I* score = 0.24).

**Figure 5.**
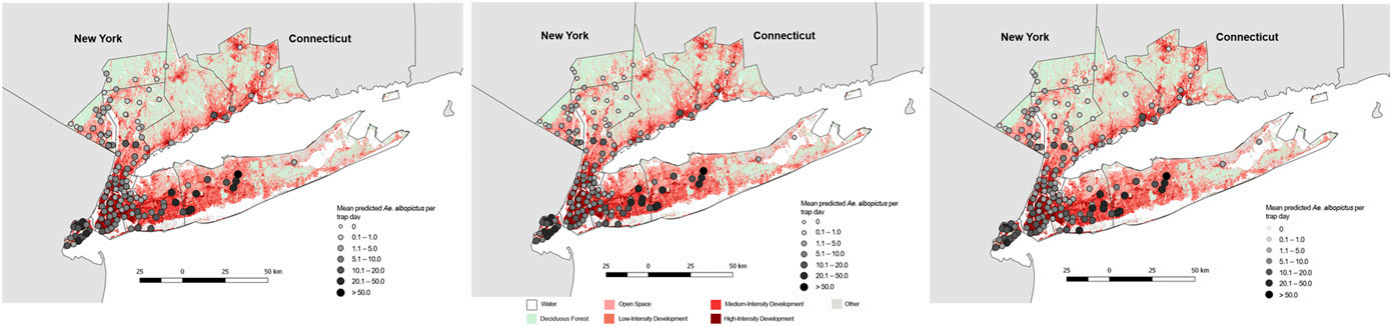
Map of predicted *Aedes albopictus* abundance across Biogents-Sentinel (BGS) traps during June–August 2017. Predicted mean *Ae. albopictus* abundance per trap day. (**A**) BGS traps active in June 2017, predictor variables for Model 4 were held at their mean values and the duration of trapping was assumed to be 24-hours. (**B**) BGS traps active in July 2017. (**C**) BGS traps active in August 2017. This figure appears in color at www.ajtmh.org.

We ran averaged model predictions for BGS trap sites during June–August and mapped the predicted abundances (Figure 5). We estimated the highest abundance detected per trap day to be in Suffolk, Nassau, and Richmond counties in NYS. Counties north of NYC showed increased detection of *Ae. albopictus* across summer months; although, by August, of the 72 sites in these counties, approximately one-third (*N* = 23) had a predicted abundance of less than one individual per trap day. Sites with a predicted abundance exceeding five individuals per trap day (*N* = 12; 16.67%) were located in southern Rockland and Westchester counties as well as within developed land cover along the Connecticut shoreline.

The RI for all variables was 1.0, with the exception of high-intensity development (*P*-value = 0.43; RI = 0.54). The RMSE of the spatially buffered LOOTCV model was 7.63, in units of mosquito abundance, indicating predictive ability within eight individuals across all observations. In addition, 57% of observations had a root of the squared prediction error falling within a value of 2.0 and 68% falling within a value of 3.0, indicating predictive ability within three individuals for a majority of observations.

## DISCUSSION

The dramatic rise of *Aedes*-transmitted diseases globally underscores the importance of mosquito surveillance and using surveillance data to understand the ecology of vectors such as *Ae. albopictus*. In 2016, there were 1,002 travel-associated imported cases of ZVD reported in NYS, making up 19% of all ZVD cases in the United States in that year.^66^ Areas with imported cases of *Aedes*-transmitted diseases and established populations of *Aedes* spp. mosquitoes, such as the northeastern United States, are at higher risk of localized virus transmission. It is, therefore, critical to understand where potential vectors exist and factors driving their abundance.

We found that landscape-level differences in temperature, precipitation, and land cover drive variation in *Ae. albopictus* abundance at the northern limit of the species distribution in NYS and Connecticut. Sites in Richmond, Nassau, and Suffolk counties had the highest abundance across summer months. Sites in more northern counties saw increased detection between June and August, likely because of population growth and dispersal from nearby urban areas; however, the overall abundance remained low (less than five individuals per trap day). We conclude that populations are well established for Long Island (Suffolk and Nassau) and NYC counties; however, north of NYC, the species is still undergoing population invasion and establishment.

Increases in mean winter temperature were highly associated with *Ae. albopictus* abundance in our study region and has been well documented in prior research.^26,27^ We hypothesized that an increase in precipitation during a time window corresponding to larval development would result in increased adult abundance. However, we found a nonlinear effect, where heavy rainfall (> 70 mm) within approximately 30 days of trapping decreases *Ae. albopictus* abundance, likely by flushing out existing larval habitat.^67,68^ This pattern has been shown for *Ae. albopictus* within forest fragments in Hawaii, while examining cumulative summer season rainfall.^66^ Results suggest that there are multiple time windows where mosquito control activities can be effective for population control. In particular, regional assessments of spring rainfall can be used to determine where *Ae. albopictus* may be highest. During the growing season, pesticide control and mosquito control outreach/communication could be conducted in the weeks following light to moderate rainfall to prevent intra-seasonal increases in the population size.

*Aedes albopictus* abundance was highest in areas with low- and medium-intensity development (composed of 20–49% and 50–79% impervious cover, respectively). However, high-intensity development (80–100% impervious cover), consisting of apartment complexes, row houses, and commercial or industrial buildings, did not have increased abundance. This may be because of greater availability of artificial breeding containers associated with peri-domestic human behaviors (e.g., lawn maintenance) in low- and medium-intensity development areas.^69^ Inconclusive results for high-intensity development may reflect a lack of artificial containers and suitable breeding habitat when vegetation and landscaping are sparse.

Investigations of *Ae. albopictus* abundance across temperate land cover types often use different definitions of “urban.” A field study in Suffolk, Rockland, and Westchester counties, NYS, found that *Ae. albopictus* abundance increases linearly from 10% to 60% impervious cover.^69,70^ Whereas a field experiment examining microclimate and *Ae. albopictus* abundance in the southeastern United States suggested that larvae in urban areas (defined as 50–100% impervious cover) experienced lower survival rates, emerged as smaller adults, and had lower growth rates than mosquitoes at suburban sites (5–50% impervious cover).^43^ These findings indicate that *Ae. albopictus* abundance may have a nonlinear association with impervious cover because of microclimate influences on mosquito fitness and/or breeding habitat availability and requires study across more nuanced gradients of urbanization than is typically conducted.^71^ For researchers who are closely examining *Ae. albopictus* along an urbanization gradient, we find it is warranted to use finer-scale cutoffs in impervious cover that are either 1) based on datasets external to NLCD (e.g., 100-m resolution impervious surface, U.S. Geological Survey) or 2) based on their own land cover classifications of remotely sensed images.

Differences in pesticide treatment may have contributed to variation in abundance demonstrated here. In 2017, 31 pesticide application events took place in four NYC counties (all except New York county). Application events included larvicide and adulticide truck spraying within residential and nonresidential areas (19 events) and aerial larviciding over marshes and natural areas (12 events). In Connecticut, there is no state-level pesticide application, and responsibility for vector control lies with local municipalities (except for disease outbreak response). Although we did not have sufficient information to quantify mosquito control, moving forward it will be important to examine how larvicidal and adulticidal treatments affect adult *Ae. albopictus* abundance over space and time.

Models 1 and 2 show that trapping methodologies have varying efficiencies for *Ae. albopictus* and emphasize the importance of accounting for such differences when using aggregated surveillance data. Whereas localized field trials have established that BGS traps are more efficient at trapping *Ae. albopictus* than CDC LTs and GTs, we show that these differences are reflected when examining empirical surveillance data.^49,72,73^ In addition, our results suggest that BGS traps with BG-Lure as well as octenol bait and/or CO2 the trapping efficiency over BG-Lure alone.^73^ We also demonstrate that CDC LTs baited with CO_2_ are almost as efficient as BGS traps with BG-Lure alone.

*Aedes albopictus* abundance is key to estimating the population-level ratio of vectors to human hosts. Given that each trap type is designed to attract mosquitoes exhibiting different behaviors, stage-specific data can be used to develop empirically based population models. Such analyses would provide more refined estimates of the vector-to-host ratio based on the abundance of host-seeking adults and could take into account the landscape and climate heterogeneity we present here. However, additional temperature-dependent and population-specific factors are necessary to determine localized vectorial capacity for autochthonous transmission, including vector competence, probability of daily mosquito survival, biting rate, and extrinsic incubation period. Field- and laboratory-based investigations of these biological parameters are ongoing in the northeastern United States and can be used to develop a more comprehensive understanding of the regional risk for *Aedes*-borne disease introduction.^74^

This investigation builds on our current understanding of *Ae. albopictus* populations in the northeast; however, several limitations exist. Foremost, mosquito populations are affected by highly localized environmental conditions. Our analysis does not consider components of microclimate and microhabitat that influence both mosquito biological rates and rates of trapping (e.g., wind velocity, shade, and vegetative detritus).^75–77^ In addition, mosquito data were integrated from seven local and state health departments, each with distinct histories of establishment, sampling design, implementation strategies, and data collection protocols. We accounted for differences in duration of trapping based on personal communication with vector control officers and estimates of sample collection procedures; however, precise information on trap deployment times were not available. *Aedes albopictus* is day-active; therefore, the hours of trap deployment may not have consistently aligned with peak mosquito activity. In addition, we found that for certain counties, global positioning system (GPS) coordinates indicated the centroid of a park or public space, whereas others documented the precise trap location. Finally, the trap sites themselves were motivated by a number of factors; the primary being a mandate to survey mosquito populations over an entire county with limited resource availability. This meant that time efficiency (e.g., distance to a central field office and placement near roads versus remote locations) played an important role in determining the trap site. As a result, these data have biases associated with convenience sampling rather than randomized placement in areas that are more fully representative of the landscape.

Despite these limitations, our findings can help guide decision-making for local mosquito control programs aiming to monitor and control *Ae. albopictus*. In particular, at a regional scale, warmer winter temperatures, increased spring precipitation, and a high proportion of medium-intensity development land cover can serve as useful indicators of where *Ae. albopictus* populations may be highest. For programs looking to monitor *Ae. albopictus* populations specifically, we recommend that BGS traps are placed in areas with low-to medium-intensity development within a 100- to 200-m buffer of the trap. However, we recommend that programs aiming to assess the distribution of multiple mosquito species across their jurisdiction place traps across the range of land cover types that are represented within their region. Training and utilization of freely available environmental datasets (e.g., NLCD and PRISM) and mapping software (e.g., QGIS) should be considered an essential component to developing robust vector surveillance systems.

County and state health departments in the United States have a long history of monitoring mosquito populations and arboviral infection status. However, surveillance and control programs have typically operated highly independently. In the short term, small modifications to current data collection practices can greatly facilitate data sharing and viability of collaborative analyses. Drop-off and pick-up time for each sampling event, GPS coordinates of the exact trap location, and trapping methodology are data elements that can be easily incorporated into data collection procedures. In the long term, it is important to recognize that decisions regarding sampling design are inherently tied to broader questions surrounding the objectives of mosquito surveillance (e.g., viral testing, species distribution to guide population control, and epidemiologic risk assessment). A better understanding of how these surveillance objectives and philosophies vary and movement toward greater consensus is crucial to maximizing the uses of these rich datasets. Such discussions are currently underway in our study region and are critical to building preparedness capacity for the emerging risk of vector-borne diseases in the United States.

## Supplemental Methods, Tables, and Figures

Supplemental materials
