# Supplementary material for: Environmental Determinants of Aedes albopictus Abundance at a Northern Limit of Its Range in the United States
Source: Am J Trop Med Hyg. 2019 Dec 12;102(2):436–47. doi: 10.4269/ajtmh.19-0244 (PMC7008348; doi:10.4269/ajtmh.19-0244)
Supplement: Supplementary file 1 [file tpmd190244.SD1.pdf]

Supplemental Material

Table S1. Land cover classes assessed at incremental spatial scales as covariates for *Aedes albopictus* abundance [a].

Buffered Distance	Open Space			Low-Intensity Development			Medium-Intensity Development			High-Intensity Development			Deciduous Forest		
	AIC	Beta	p-value	AIC	Beta	p-value	AIC	Beta	p-value	AIC	Beta	p-value	AIC	Beta	p-value
**100**	35867	-0.11	<0.001	35400*	0.52	<0.001	35232	0.59	<0.001	35852	0.15	<0.001	35511	-0.47	<0.001
**200**	35833	-0.17	<0.001	35483	0.47	<0.001	35340*	0.64	<0.001	35834	0.19	<0.001	35319	-0.61	<0.001
**300**	35812*	-0.19	<0.001	35515	0.44	<0.001	35515	0.44	<0.001	35846	0.16	<0.001	35180	-0.70	<0.001
**400**	35822	-0.18	<0.001	35543	0.42	<0.001	35543	0.42	<0.001	35850	0.16	<0.001	35041	-0.78	<0.001
**500**	35821	-0.17	<0.001	35500	0.42	<0.001	35550	0.42	<0.001	35838	0.18	<0.001	34963*	-0.82	<0.001

This table presents results of bivariate negative binomial logistic regression analyses for the percent of land cover class at buffered distances around each trap coordinate in association with *Ae. albopictus* abundance. Model results with the lowest Akaike Information Criterion (AIC) are highlighted in grey and were selected for evaluation in multivariable analyses. Scaled Beta regression coefficients (Beta values) indicate that Deciduous Forest, Medium Intensity Development, and Low Intensity Development had the strongest association with *Ae. albopictus* abundance. Land cover classes are based on the 2011 National Land Cover Database.

[a] Predictors (indicated x) were scaled using their mean and standard deviation (sd) using the following equation: $[x - \text{mean}(x)] / [\text{sd}(x)]$.

Table S2. Landscape drivers of *Aedes albopictus* abundance.

Model	Predictor Variables	Scaled Coefficient Estimate	95% CI	p-value	AIC	Weight
3A	Intercept	-2.89	-2.98, -2.81	< 0.01	33498	0.51
	Autocovariate [a]	0.32	0.29, 0.34	< 0.01		
	Gravid Trap (GT)	-1.30	-1.42, -1.10	< 0.01		
	CDC Light Trap (LT)	-0.86	-0.96, -0.77	< 0.01		
	Open Space: 300-meter buffer	-0.09	-0.14, 0.04	< 0.01		
	Low-intensity Development: 100-meter buffer	0.20	0.16, 0.25	< 0.01		
	Medium-intensity Development: 200-meter buffer	0.27	0.21, 0.33	< 0.01		
	Deciduous Forest: 500-meter buffer	-0.39	-0.44, -0.33	< 0.01		
	Road Density	-0.59	-0.73, -0.46	< 0.01		
	Road Density^2	0.64	0.51, 0.79	< 0.01		
3B	Intercept	-2.90	-2.98, -2.81	< 0.01	33499	0.49
	Autocovariate [a]	0.32	0.30, 0.34	< 0.01		
	Gravid Trap (GT)	-1.30	-1.41, -1.18	< 0.01		
	CDC Light Trap (LT)	-0.87	-0.96, -0.77	< 0.01		
	Open Space: 300-meter buffer	-0.07	-0.13, 0.01	0.01		
	Low-intensity Development: 100-meter buffer	0.22	0.16, 0.26	< 0.01		
	Medium-intensity Development: 200-meter buffer	0.28	0.22, 0.33	< 0.01		
	High-intensity Development: 200-meter buffer	0.03	-0.01, 0.08	0.16		
	Deciduous Forest: 500-meter buffer	-0.37	-0.43, -0.31	< 0.01		
	Road Density	-0.60	-0.73, -0.46	< 0.01		
	Road Density^2	0.65	0.51, 0.79	< 0.01		
3A–3B	**Total AIC Weight**					1.00

This table presents the best-performing models (ΔAIC < 2) from the 256 models that included all combinations of landscape variables. The 95% Confidence Interval (CI) of the estimates indicate an effect on the detection of *Ae. albopictus* when the CI does not include zero (p-value <0.05). The weight of the model describes the relative contribution of each model to the multi-model inferred averaged model (Table 3).

[a] Autocovariate term is defined using distance-based neighbors, where the upper distance bound is set to 17,073 meters. This value is derived from the semivariogram model of Model 3 residuals (run with only landscape variables and without an autocovariate term).

Table S3. Landscape and meteorological drivers of *Aedes albopictus* abundance.

Model	Predictor Variables	Scaled Coefficient Estimate	95% CI	p-value	AIC	Weight
4A	Intercept	-2.76	-2.86, -2.67	< 0.01	33146	0.54
	Autocovariate	0.26	0.24, 0.29	< 0.01		
	Gravid Trap (GT)	-1.38	-1.50, -1.27	< 0.01		
	CDC Light Trap (LT)	-0.87	-0.96, -0.77	< 0.01		
	Open Space: 300-meter buffer	-0.13	-0.17, -0.08	< 0.01		
	Low-intensity Development: 100-meter buffer	0.17	0.12, 0.21	< 0.01		
	Medium-intensity Development: 200-meter buffer	0.26	0.20, 0.31	< 0.01		
	Deciduous Forest: 500-meter buffer	-0.36	-0.43, -0.30	< 0.01		
	Road Density	-0.62	-0.75, -0.50	< 0.01		
	Road Density^2	0.63	0.49, 0.76	< 0.01		
	Minimum Winter Temperature	0.19	0.12, 0.27	< 0.01		
	March Precipitation	0.26	0.21, 0.32	< 0.01		
	Lagged Precipitation	1.29	1.11, 1.47	< 0.01		
	Lagged Precipitation ^{2}	-1.56	-1.75, -1.37	< 0.01		
4B	Intercept	-2.77	-2.86, -2.67	< 0.01	33146	0.46
	Autocovariate	0.26	0.25, 0.29	< 0.01		
	Gravid Trap (GT)	-1.38	-1.49, -1.27	< 0.01		
	CDC Light Trap (LT)	-0.86	-0.96, -0.77	< 0.01		
	Open Space: 300-meter buffer	-0.11	-0.17, -0.05	< 0.01		
	Low-intensity Development: 100-meter buffer	0.17	0.13, 0.22	< 0.01		
	Medium-intensity Development: 200-meter buffer	0.26	0.20, 0.32	< 0.01		
	Hight-intensity Development: 200-meter buffer	0.04	-0.01, 0.09	0.12		
	Deciduous Forest: 500-meter buffer	-0.35	-0.41, -0.28	< 0.01		
	Road Density	-0.63	-0.76, -0.59	< 0.01		
	Road Density^2	0.63	0.50, 0.76	< 0.01		
	Minimum Winter Temperature	0.18	0.10, 0.26	< 0.01		
	March Precipitation	0.26	0.21, 0.32	< 0.01		
	Lagged Precipitation	1.29	1.10, 1.47	< 0.01		
	Lagged Precipitation ^{2}	-1.56	-1.75, -1.36	< 0.01		
4A–4B	**Total AIC Weight**					1.00

This table presents the best-performing models (ΔAIC < 2) from the 4096 models that included all combinations of landscape and meteorological variables. The 95% Confidence Interval (CI) of the estimates indicate an effect on the detection of *Ae. albopictus* when the CI does not include zero (p-value <0.05). The weight of the model describes the relative contribution of each model to the multi-model inferred averaged model (Table 4).

[a] Autocovariate term is defined using distance-based neighbors, where the upper distance bound is set to 17,385 meters. This value is derived from the semivariogram model of Model 4 residuals (run with only landscape and meteorological variables and without an autocovariate term).

Figure S1. Land cover characteristics for counties and within a 500-meter buffer of trap sites.

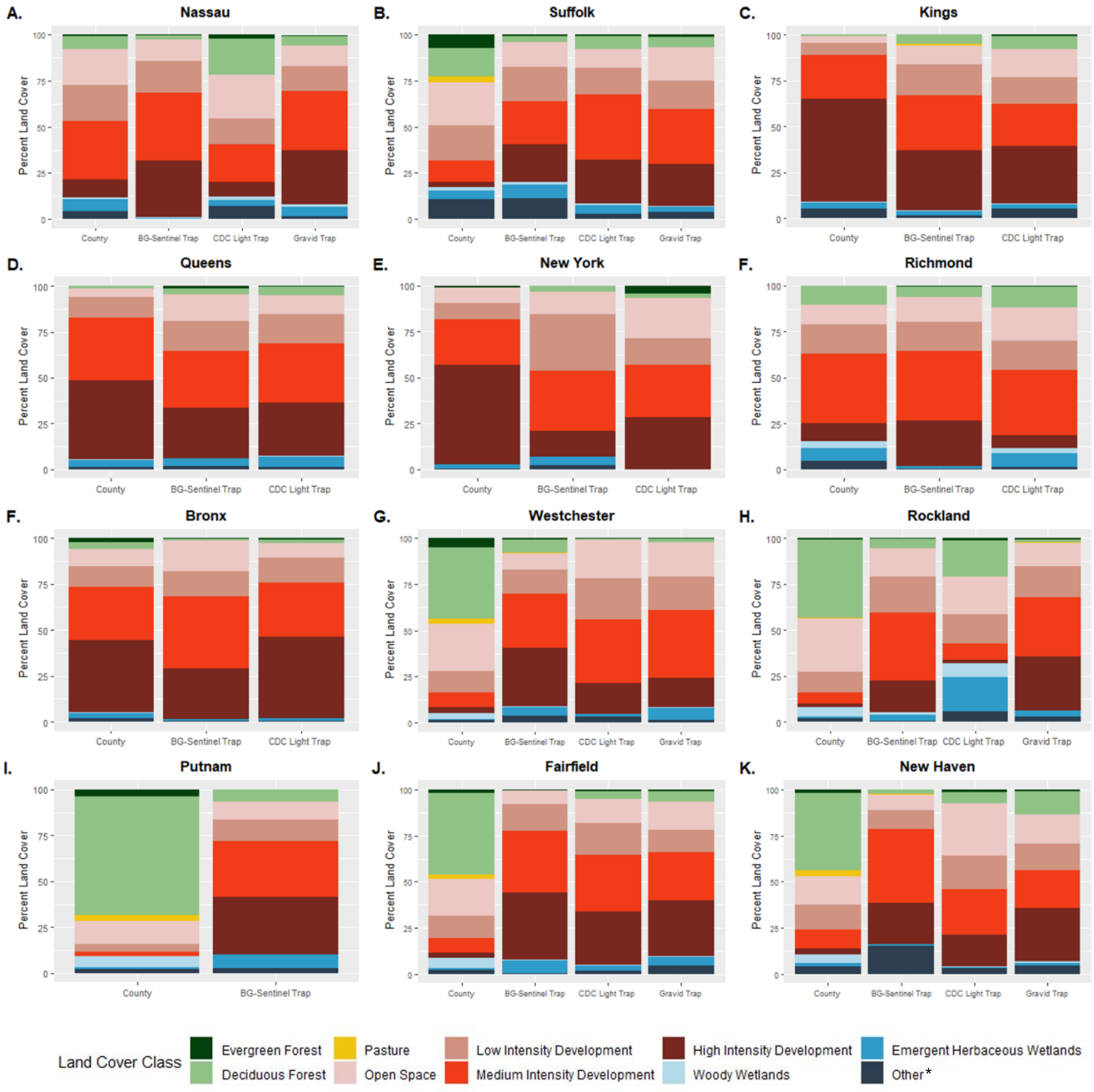

Land cover class composition for 12 counties in New York State and Connecticut. Each graph shows the percent land cover for the county overall and the mean percent land cover within a 500-meter buffer for all traps in the county (excluding water classes). Here we visually assess whether trap placement may be representative of the overall county land cover composition. Land cover classes are based on the 2011 National Land Cover Database.

* Other classification included: Barren Land, Mixed Forest, Shrub/Scrub, Grassland/Herbaceous, and Cultivated Crops.

Supplemental Material

Methods

Model Development

We assessed the AIC for each candidate model using Equation S1, where K is the number of parameters in the model. The preferred candidate model has the lowest AIC, balancing model fit with parsimony.

$$AIC = -2\log(Likelihood) + 2K \qquad \text{Equation S1}$$

We then used a model averaging procedure, to incorporate information across multiple top-ranked models, and calculated Akaike weights (wAIC) for each model (Equation S2). Here Δ_i is the difference in AIC values between the most parsimonious candidate model and model i ($\Delta_i = \text{AIC}_i - min\text{AIC}$), given a total set of m candidate models. The wAIC is a value between 0 and 1, with the sum of all wAICs for a set of m candidate models being equal to 1.

$$w_i = \frac{\exp\left[-\frac{1}{2}\Delta_i\right]}{\sum_{i=1}^{m}\exp[-\frac{1}{2}\Delta_i]} \qquad \text{Equation S2}$$

We calculated averaged parameter (Equation S3) and standard error (SE) (Equation S4) estimates across top candidate models using a "natural averaging" approach, where parameters were averaged only for covariates in the best-performing models (ΔAIC < 2) [4]. The averaged parameter estimate and corresponding SE were calculated such that $\widehat{\beta_i}$ is the estimate for the predictor and w_i is the wAIC for the model i [5]. We calculated the $\hat{\hat{\beta}}$ and $\widehat{SE}\left(\hat{\hat{\beta}}\right)$ only for the models in which the predictor of interest appears (R, package 'MuMin').

$$\hat{\hat{\beta}} = \frac{\sum_{i=1}^{R} w_i\widehat{\beta_i}}{\sum_{i=1}^{R} w_i} \qquad \text{Equation S3}$$

$$\widehat{SE}(\hat{\hat{\beta}}) = \sum w_i\sqrt{\widehat{var}(\widehat{\beta_i}) + (\widehat{\beta_i} - \hat{\hat{\beta}})^2} \qquad \text{Equation S4}$$

Spatial Autocorrelation

Global Moran's I

This analysis allowed us to compare whether patterns in *Ae. albopictus* abundance are significantly clustered, dispersed or random. Analyses derived a global Moran's I value across the 338 trap sites using Equation S5. [3] In this computation, pairs of trap sites are represented by i and j, where $z(x_i)$ and z_i is the deviation in the model residual for trap site i from its average over all the sites ($x_i - \bar{x}$). This deviation is calculated for all neighboring features within the range of spatial autocorrelation and multiplied together to create a cross-product. Here, w_{ij} is an assigned weight dependent on the distance between trap sites i and j, and S_o is the aggregate of all spatial weights. If abundance values are shown to cluster in space, the I index will be positive, and when they are highly dispersed the I index will be negative. The analysis then calculates expected I value. The expected and observed indices are compared, and the package computes a z-score and p-value indicating whether the difference is statistically significant (R, package 'ape').

$$I = \frac{n\sum_{i=1}^{n}\sum_{j=1}^{n} w_{i,j}z_iz_j}{S_0\sum_{i=1}^{n} z_i^2} \qquad \text{Equation S5}$$

References

1. Matheron G, 1963. Principles of geostatistics. Econ Geol, 58: 1246–1266.
2. Clark I, 1979. Practical Geostatistics. London, United Kingdom: Applied Science Pub.
3. Griffith DA, 1987. Spatial autocorrelation: A primer resource publications in geography. Washington, DC: Association of American Geographers.
4. Burnham KP, Anderson DR, 2003. Model selection and multimodel inference: a practical information-theoretic approach. Berlin, Germany: Springer Science & Business Media.
5. Symonds MRE, Moussalli A, 2011. A brief guide to model selection, multimodel inference and model averaging in behavioural ecology using Akaike's information criterion. Behav Ecol Sociobiol 65: 13–21.
